# Digital Engagement Phenotypes and 6-Month Weight Loss in a Tirzepatide-Supported Digital Weight-Loss Program: A Retrospective Cohort Study

**DOI:** 10.3390/medicina62091647

**Published:** 2026-08-27

**Authors:** Louis Talay, Connie Xu, Jason Hom, Laura Swinckels, Marilyn Tan, John Alderete, Neera Ahuja

**Affiliations:** 1 Eucalyptus Health, Sydney 2000, Australia; laura.swinckels@eucalyptus.health; 2 Amigo AI, New York, NY 10010, USA; connie@amigo.ai; 3 Department of Medicine, Stanford University Medical Centre, Stanford, CA 94305, USA; jasonhom@stanford.edu (J.H.); mjtan@stanford.edu (M.T.); nkahuja@stanford.edu (N.A.); 4 Department of Linguistics, Simon Fraser University, Burnaby, BC V5A 1S6, Canada; john.alderete@gmail.com

**Keywords:** real-world obesity service, tirzepatide, digital weight-loss service, program engagement, program perseverance

## Abstract

*Background and Objectives*: Real-world persistence with medication-supported weight management programs is often low. While digital weight loss services (DWLS) provide multi-modal digital supports to improve engagement and counter attrition, the existing literature frequently relies on unidimensional or binary classifications of user engagement. This study used unsupervised machine learning to identify distinct digital engagement phenotypes and evaluated their independent associations with 6-month weight loss outcomes in patients prescribed tirzepatide. *Materials and Methods*: This retrospective cohort study analyzed deidentified data from 9470 medication-adherent, complete-case adult patients (out of 39,220 tirzepatide initiators) within a British DWLS who initiated tirzepatide between 20 May 20 and 2 December 2025. *K*-means clustering was performed on four continuous, longitudinal usage metrics: weekly app logins, health coach messaging, automated assistant (JuneBot) messaging, and weight tracking. To evaluate the primary clinical endpoint—6-month percentage weight loss—unadjusted pairwise comparisons (Tukey HSD) and a fully adjusted ordinary least squares multivariate linear regression model were executed to control for baseline demographic, clinical, and interim behavioral covariates. *Results*: Four stable engagement phenotypes emerged: non-engaged (*n* = 1772), high health coach engagement (n = 1141), high JuneBot engagement (n = 1472), and passive self-trackers (n = 5085). In a baseline-adjusted multivariate regression model, all active phenotypes were independently associated with 6-month weight loss relative to the low engagement baseline. Compared with this reference group, adjusted mean differences in percentage weight loss were 4.60% (SE = 0.26, *p* < 0.001) in the high health coach cluster, 4.69% (SE = 0.24, *p* < 0.001) in the high JuneBot cluster, and 3.71% (SE = 0.19, *p* < 0.001) in the passive self-tracker cluster. *Conclusions*: In this selected per-protocol, complete-case cohort of patients who persisted with tirzepatide treatment and reported 6-month weight data, distinct patterns of digital engagement were associated with different weight-loss outcomes. Greater conversational and self-tracking engagement was associated with greater observed 6-month weight loss than low engagement after adjustment for measured baseline characteristics. However, the retrospective observational design, concurrent assessment of engagement and outcome, substantial cohort selection, and potential residual confounding preclude causal or comparative-effectiveness conclusions, including claims of equivalence between automated and human support. Prospective studies with temporally defined engagement exposures and randomized allocation to support modalities are required.

## 1. Introduction

Obesity represents one of the most pervasive and severe global health challenges of the 21st century, deeply impacting both public health infrastructure and individual life expectancy. Chronic weight-related conditions—including type 2 diabetes, cardiovascular disease, metabolic dysfunction-associated steatotic liver disease, and various malignancies—contribute significantly to global mortality and place immense financial strain on healthcare systems worldwide [1]. According to recent World Health Organization (WHO) estimates, more than 1 billion individuals globally are living with obesity, a figure that continues to rise across virtually every demographic sector [2]. For decades, clinical interventions relied heavily on behaviorally driven lifestyle modifications; however, long-term weight loss maintenance through willpower and caloric restriction alone has historically yielded low success rates because of complex physiological and metabolic counter-regulatory mechanisms that resist weight reduction [3].

The recent emergence and widespread adoption of glucagon-like peptide-1 receptor agonists (GLP-1 RAs) and dual GIP/GLP-1 receptor agonists, such as tirzepatide, has fundamentally transformed the chronic weight management landscape. These pharmacological therapies provide highly effective, scalable solutions by mimicking incretin hormones to enhance satiety, delay gastric emptying, and regulate central appetite pathways [4], resulting in unprecedented double-digit percentage weight loss in clinical trials [5]. Nevertheless, international health institutions, including the WHO and the National Institute for Health and Care Excellence (NICE), explicitly stress that these medications are not standalone cures. Instead, clinical guidelines dictate that GLP-1 RAs should strictly serve as adjuncts to continuous, comprehensive obesity care, which includes structured behavioral counseling, nutritional restructuring, and physical activity tracking to preserve lean mass and prevent rapid weight regain upon treatment cessation [6].

Despite this clear clinical consensus, delivering continuous, comprehensive obesity care remains exceedingly difficult in traditional face-to-face medical settings. Patients frequently struggle to adhere to intensive lifestyle modification programs due to systemic barriers such as geographic remoteness, high financial costs, limited practitioner availability, and the social stigma often encountered in physical clinic environments. As a result, attrition rates in conventional behavioral interventions remain high, leaving a critical gap in multidisciplinary support during pharmacological treatment [7].

To overcome these physical limitations, app-mediated Digital Weight Loss Services (DWLS) have expanded rapidly, significantly improving patient access to remote multidisciplinary teams (MDTs) and structured educational content [6,8]. However, digital health critics argue that simply providing access to a mobile application does not guarantee active patient engagement or continuity of care. Because digital platforms inherently rely on user initiative, large portions of patient cohorts can default to passive usage or disengage entirely, potentially undermining the long-term efficacy of the supported medical therapy [9].

Consequently, healthcare scholars have begun investigating the explicit relationship between digital application engagement patterns and clinical weight loss outcomes [10,11]. Importantly, greater application use is not universally beneficial and may also reflect greater need, treatment difficulty, or distress rather than purely effective intervention exposure. Engagement with the digital platform is also distinct from adherence to medical, nutritional, or physical-activity recommendations, and these dimensions do not always move in parallel [8]. While these early publications have consistently found that “more engagement” is associated with better outcomes, they typically operationalise engagement using coarse, often binary or single-metric constructs—for example, classifying patients as “engaged” versus “non-engaged” based on a minimum app-use threshold. This approach is not unique to weight-loss programs: similar binary or unidimensional engagement markers are widely used across digital mental-health, diabetes self-management, and remote cardiac-rehabilitation interventions. Such simplifications obscure the fact that modern digital platforms support multiple, overlapping engagement modalities—including passive biometric self-tracking, asynchronous human coaching, automated conversational agents, and educational content consumption—that may contribute differently to outcomes. A more nuanced understanding of these multi-modal engagement patterns is needed to inform how best to design and allocate scalable digital supports alongside pharmacologic therapy.

To directly address the specific methodological gap presented by the binary classification used in the recent literature, this study uses unsupervised clustering to derive naturally occurring digital engagement phenotypes and then examines how these patterns relate to weight-loss outcomes. By shifting from a static binary to a multidimensional analysis, we aim to map the naturally occurring, nuanced behavioral trajectories that emerge when patients interact with diverse digital resources. The primary objective of this study is to evaluate the effect of distinct digital engagement phenotypes on 6-month percentage weight loss in a large per-protocol cohort of DWLS patients receiving tirzepatide treatment. Through this approach, we seek to determine the extent to which a highly scalable, rule-based AI framework can support patients in a comprehensive medicated DWLS.

## 2. Materials and Methods

### 2.1. Study Design and Patient Population

This study adopted a retrospective cohort design to evaluate the relationship between distinct digital engagement phenotypes and 6-month weight-loss outcomes among patients utilizing an app-mediated DWLS. The primary objective was to examine the effect of different longitudinal engagement modalities on 6-month percentage weight loss supported by tirzepatide treatment. Investigators followed the Strengthening the Reporting of Observational Studies in Epidemiology (STROBE) guidelines throughout the study design and reporting phases [12].

To achieve the study objectives, investigators filtered for patients who persisted with the program over 6 months (Per Protocol). Patients were included in the final analysis if they met the following criteria:

1. They initiated treatment between May 20 and December 2 2025. The former date ensured access to all active automated and human coaching features (Junebot was launched on 20 May 2025), while the latter was exactly 6 months prior to data extraction (2 June 2026) and enabled all patients the chance to submit follow-up weight data.
2. They received a minimum of 5 Tirzepatide medication orders within a 183-day (6-month) observation window to ensure adequate pharmacological exposure. This serves as a proxy for medication persistence as data on whether patients actually administered the medication were not available.
3. They did not switch from Tirzepatide to any other weight-loss medication during their first 183 days (e.g., Semaglutide), to ensure their clinical trajectories remained specific to Tirzepatide.
4. They provided a verified body weight entry within a strict 6-month clinical window, defined as 173 to 193 days post-program initiation.

All study data were stored in the Juniper central data repository on Google BigQuery, a serverless, cloud-based warehouse. Data were extracted by investigators via the SQL language. Weight data were recorded either via Bluetooth-connected scales supplied by the program or via manual self-entry in the app. A team of Juniper health coaches verify weight data plausibility on a weekly basis. Investigators also implemented hard exclusion criteria for all 6-month values ≥ and ≤30% of baseline weight. No distinction was made between Bluetooth-connected scales or self-uploaded values. The Stanford University Independent Research Board determined that the study did not meet the definition of human subject research as defined in federal regulations 45 CFR 46.102 or 21 CFR 50.3 (Protocol 82970, 22 October 2025).

### 2.2. Program Overview

The Juniper UK DWLS serves adults with overweight or obesity (BMI ≥ 25) and operates as an asynchronous, GLP-1 RA-supported clinical program designed for chronic weight management. The journey begins with a comprehensive digital triage in which prospective patients submit a pre-consultation questionnaire covering baseline health markers. These data are reviewed by a pharmacist-independent prescriber to assess whether the patient meets the clinical criteria for pharmacological intervention. Patients are eligible for tirzepatide prescription if they have a BMI of ≥30 kg/m^2^, or a BMI of 27–29.9 kg/m^2^ in the presence of at least one weight-related comorbidity (National Institute for Health and Care Excellence, 2024), with off-label prescribing considered at the clinical discretion of the prescriber for patients below these thresholds. They also detail absolute contraindications for tirzepatide-supported treatment, which include acute kidney disease, acute pancreatitis, hypoglycaemia, severe gastrointestinal disease, multiple endocrine neoplasia syndrome type 2, a personal or family history of medullary thyroid cancer, and a known sensitivity to tirzepatide or any of the product’s components [13].

Patients who are deemed eligible are then asked to pay a monthly subscription fee. Over the study period, first month fees ranged from 189 to 279 Great Britain Pounds (GBP), and highest fees (for higher tirzepatide doses) ranged from 294 to 339 GBP. Upon completion of the first monthly payment, patients receive access to the Juniper UK application, which includes educational content, a bluetooth-connected weight tracking tool, and unlimited communication with a multidisciplinary (MDT) care team. MDTs consist of a prescribing practitioner, a university-qualified health coach (nutritionist or dietitian), and a medical support officer. Health coaches send patients a fortnightly check-in message unless patients communicate at a higher frequency. Educational content is multi-modal, structured around core metabolic pillars including sustainable caloric deficits, macronutrient/protein targets, and physical movement tracking. Adverse events are self-reported via the app using a severity-rating scale, where moderate events trigger ad hoc medical consultations and severe events trigger immediate escalation protocols. All patient communications, tracking logs, and questionnaire responses are stored in Juniper’s central data repository.

### 2.3. JuneBot Functionality

Research In addition to human coaching, the Juniper digital ecosystem features an automated conversational assistant named JuneBot. During the study period, it used a rule-based decision framework with natural-language classification and templated responses drawn from a curated guideline repository, and it did not employ generative large language models. Rather than executing automated changes or modifying a patient’s active clinical record, it operates purely as an empathetic, non-judgmental information resource and conversational support tool designed to reinforce the program’s baseline programmatic guidelines. A previous study was dedicated to testing the safety of the agent’s communication within the Juniper program [14].

To maintain strict clinical safety boundaries, explicit operational guardrails govern the JuneBot interaction matrix:

- No Medical Advice: JuneBot is prohibited from delivering personalized clinical assessments, diagnostic evaluations, or prescription adjustments. It only dispenses global, established knowledge bases and pre-approved clinical guidelines.
- Non-Agentic Framework: The assistant cannot act on behalf of the patient, manipulate medication order frequencies, or execute system-level billing or programmatic operations within the application.
- No MDT Substitution: JuneBot serves purely as a programmatic augment and is structurally prevented from replacing a practitioner, pharmacist, or university-qualified health coach.
- Mental Health Guardrails: The system does not manage or process mental health protocols. Conversational inputs that flag psychological distress, severe body dysmorphia, or crises bypass the automated interface entirely and trigger an immediate, mandatory human escalation protocol to the clinical support team.

JuneBot’s informational repository is partitioned into specific behavioral and programmatic domains, including diet, nutrition, and eating behavior; exercise and physical movement; weight loss tracking dynamics; and standardized application/technical support. Regarding conversation frequency, JuneBot operates primarily on an opt-in engagement model, initiating conversational sequences only after a patient logs an initial conversational input. This user-initiated framework is supplemented by a structured longitudinal engagement protocol consisting of a low-frequency, automated weekly “nudge” designed to establish continuous behavioral reinforcement without inducing digital communication fatigue.

### 2.4. Statistical Analysis and Cluster Generation

An unsupervised machine learning approach was implemented using K-means clustering [15] to discover naturally occurring behavioral archetypes without imposing investigator-defined cut-offs. The clustering algorithm was trained on four continuous behavioral percentage metrics captured longitudinally from program initiation up to the 6-month endpoint:

- Weekly app engagement percentage: The proportion of weeks a patient actively logged into the mobile application interface.
- Weekly health coach messaged percentage: The proportion of weeks a patient initiated or replied to conversational messages with a university-qualified human health coach.
- Weekly JuneBot messaged percentage: The proportion of weeks a patient initiated or replied to conversational messages with JuneBot.
- Weekly weight track percentage: The proportion of weeks a patient tracked their weight via the Juniper bluetooth scales.

### 2.5. Mathematical Preprocessing and Optimization

Because K-means clustering is highly sensitive to variances in variable scaling, all four continuous engagement metrics were standardized via Z-score transformation (μ = 0, σ = 1) prior to distance matrix calculation. Missing data points within these critical fields were handled via listwise deletion to ensure complete cases for the clustering algorithm.

To identify a suitable number of clusters (K), the scaled data were evaluated across a search space of 1 ≤ K ≤ 10 using two distinct metrics: the Elbow Method (tracking total within-cluster sum of squares [WSS]) and the Average Silhouette Method [16]. The silhouette width was maximised at K = 2, suggesting a coarse division between low- and high-engagement users, whereas the WSS curve continued to decrease steeply up to approximately K = 4 before flattening. Because K = 2 merged conceptually distinct engagement modes (for example, conversational versus predominantly passive tracking) into a single “engaged” class, we selected candidate solutions in the range K = 3–5 and prioritised those that yielded clinically interpretable phenotypes.

To further assess the robustness of the 4-cluster solution, we computed the gap statistic across K = 1–10 and conducted bootstrap cluster-stability analyses. The gap statistic increased sharply from K = 1 to K = 4–5, with only modest gains beyond this range, and identified K = 5 as the optimal value under the Tibshirani et al. criterion (Supplementary Figure S1, Table S1). Thus, formal criteria did not converge on a single K value (silhouette K = 2, gap K = 5). We therefore focused on solutions in the range K = 3–5 and selected K = 4 as a parsimonious compromise within the gap-statistic plateau that separated a low-engagement group from two distinct conversationally engaged groups and a predominantly self-tracking group, which we judged to be clinically interpretable. We retained the 4-cluster solution for the main analyses. For cluster-stability assessment, we performed bootstrap resampling with B = 100. In each bootstrap sample, we re-ran the full K-means procedure at K = 4 using the same initialization settings (multiple random starts and maximum iterations) as in the original sample. Clusters from each bootstrap run were matched to the original clusters by maximizing the Jaccard similarity of membership sets. We then computed the mean Jaccard index across resamples for each cluster, which yielded values of 0.99, 0.99, 0.99, and 0.98 (Supplementary Table S2), indicating very high reassignment stability and little evidence of fragmentation or merging. Because the engagement metrics are bounded percentages with relatively well-separated modes, such high stability values are plausible, although the bounded nature of the data may also contribute to tighter cluster cores.

For visualisation purposes only, we then projected the four-dimensional engagement metrics onto the first two principal components, which together explained 81.2% of the variance; this projection is presented in the Results to illustrate the geometric separation of the clusters. Because K-means clustering is highly sensitive to variance in variable scaling, all four continuous engagement metrics were standardized via Z-score transformation (μ = 0, σ = 1) prior to distance matrix calculation.

### 2.6. Prototype Profiling and Nominal Variable Generation

Following cluster generation, individual patient cluster assignments were extracted as discrete nominal factors and merged back into the primary unscaled dataset. For clinical interpretation, we assigned descriptive labels based on dominant engagement patterns across the four metrics. One cluster exhibited minimal app use, messaging activity, and weight tracking and was labelled “non-engaged.” A second showed high app use, frequent weight tracking, and frequent messaging with human health coaches (“high health coach engagement”). A third had similarly high app and tracking activity but predominantly interacted via the automated assistant rather than human coaches (“high JuneBot engagement”). The fourth cluster combined high app use and consistent weight tracking with minimal conversational messaging (“passive self-trackers”). These nominal cluster assignments served as the primary exposure variables for all downstream clinical endpoint evaluations and are described quantitatively in the Results.

### 2.7. Statistical Analysis

The primary clinical endpoint was the observed 6-month percentage weight loss, defined as the percentage change in body weight between program initiation and the closest verified weight measurement within a strict 6-month follow-up window (days 173–193 after initiation). Descriptive statistics for this clinical endpoint were stratified across the four behavioral clusters and summarized as means with standard deviations (SD).

To examine the effect of each engagement prototype on 6-month weight loss, a multivariate ordinary least squares linear regression model was constructed. The primary independent variable of interest was the 4-category cluster assignment nominal factor, with the non-engaged group designated as the reference category.

To reduce confounding from baseline and interim differences, the regression model adjusted for various demographic and clinical covariates. Demographic variables included age, ethnicity and sex at birth. Clinical markers were baseline BMI, comorbidity count, and GLP-1 RA use in the 6 months prior to program start. A second multivariate linear regression model was used to assess the effect of post-baseline variables (in addition to the model 1 variables), including first month weight-track count, weight-loss plateau, side effect incidence (yes/no), maximum tirzepatide dose, and clinically significant weight loss (≥5%) within the first 3 months of treatment.

Unstandardized regression coefficients with corresponding 95% confidence intervals (CI) were calculated for each engagement prototype to represent the incremental percentage weight loss achieved relative to the non-engaged baseline. The investigators assessed multicollinearity using variance inflation factors (VIFs), with VIF > 10 indicating potential concern.

All statistical pipelines and data visualizations were executed within RStudio (version 2023.06.1). Statistical significance for all downstream analyses was maintained at a two-tailed α = 0.05.

## 3. Results

**Figure 1:**
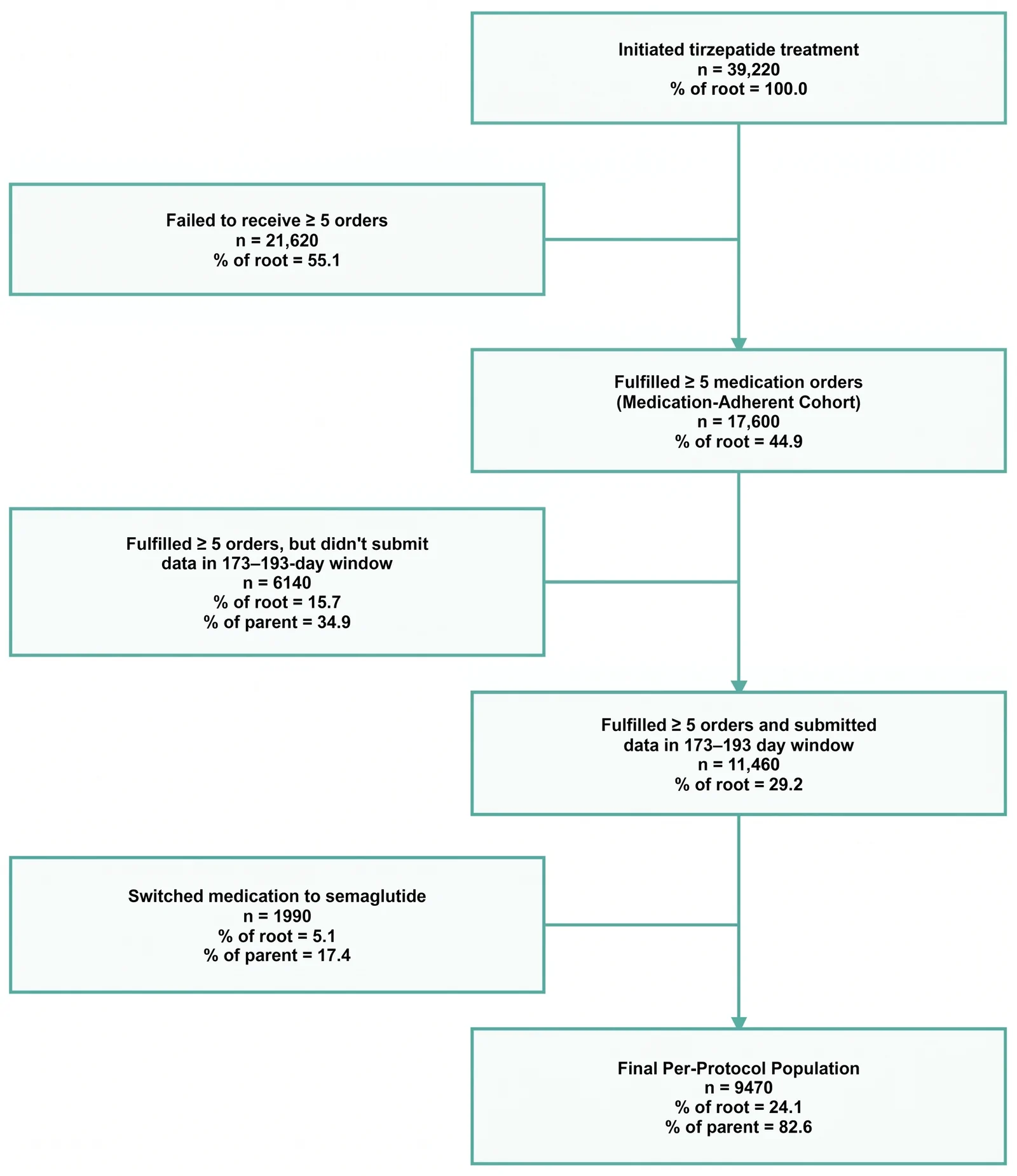
Patient flow chart.

A total of 39,220 patients initiated tirzepatide treatment within the specified study window. Of these, 17,600 patients met the baseline per-protocol criteria for medication persistence fulfilling a minimum of 5 medication orders by 183 days post-program initiation. Within this medication-adherent cohort, 11,460 patients successfully provided a verified 6-month body weight entry within the strict day 173–193 clinical follow-up window. A further 1990 patients were omitted from the final analysis matrix due to having changed medication to semaglutide, yielding a final, complete-case study population of 9470 patients for behavioral cluster generation and outcome modelling (Figure 1).

The baseline demographic and clinical profiles of the 9470 patients were stratified across the four emergent *K*-means engagement clusters (Table 1). Pearson’s Chi-squared tests of independence demonstrated that all baseline characteristics varied significantly across the four behavioral phenotypes (*p* < 0.001). Notably, cluster 2 (high health coach engagement) exhibited the highest concentration of female patients (94%), individuals presenting with a baseline BMI ≥ 40 kg/m^2^ (22%), and patients managing a complex comorbidity burden of 3 or more baseline conditions (25%). Conversely, cluster 1 (non-engaged) featured a higher baseline proportion of male patients (27%) and individuals in lower BMI tiers compared to the highly interactive cohorts.

### 3.1. Engagement Cluster Profiles

The unsupervised K-means clustering algorithm generated distinct behavioral phenotypes based on the four continuous tracking metrics. The average behavioral profiles mapping to each cluster are detailed in Table 2.

- Cluster 1 (n = 1772): non-engaged patients, displaying pervasively low interaction across all app modalities, including a baseline weekly app engagement rate of 30.0% and nominal tracking or messaging behaviors.
- Cluster 2 (n = 1141): patients with high health coach enagagement, demonstrating high baseline tracking metrics combined with a high propensity to communicate through the human coaching channel (50.9% of weeks) over automated alternatives (22.4%).
- Cluster 3 (n = 1472): patients with high JuneBot engagement, displaying identical app and tracking frequencies to Cluster 2, but substituting human engagement for dominant interaction with the automated AI interface (46.2% of weeks).
- Cluster 4 (n = 5085): passive self-trackers, representing the largest cohort. These users demonstrated high baseline app utilization (86.7%) and consistent biometric weight logging (78.8%) but remained largely silent across human and automated messaging modules.

Each cluster reflects a dominant engagement pattern but includes meaningful use of other modalities (e.g., high JuneBot users also interacted with human coaches to some extent). Clusters should not be interpreted as exposure to mutually exclusive interventions. Median and IQR distributions are presented in Supplementary Table S3.

**Figure 2:**
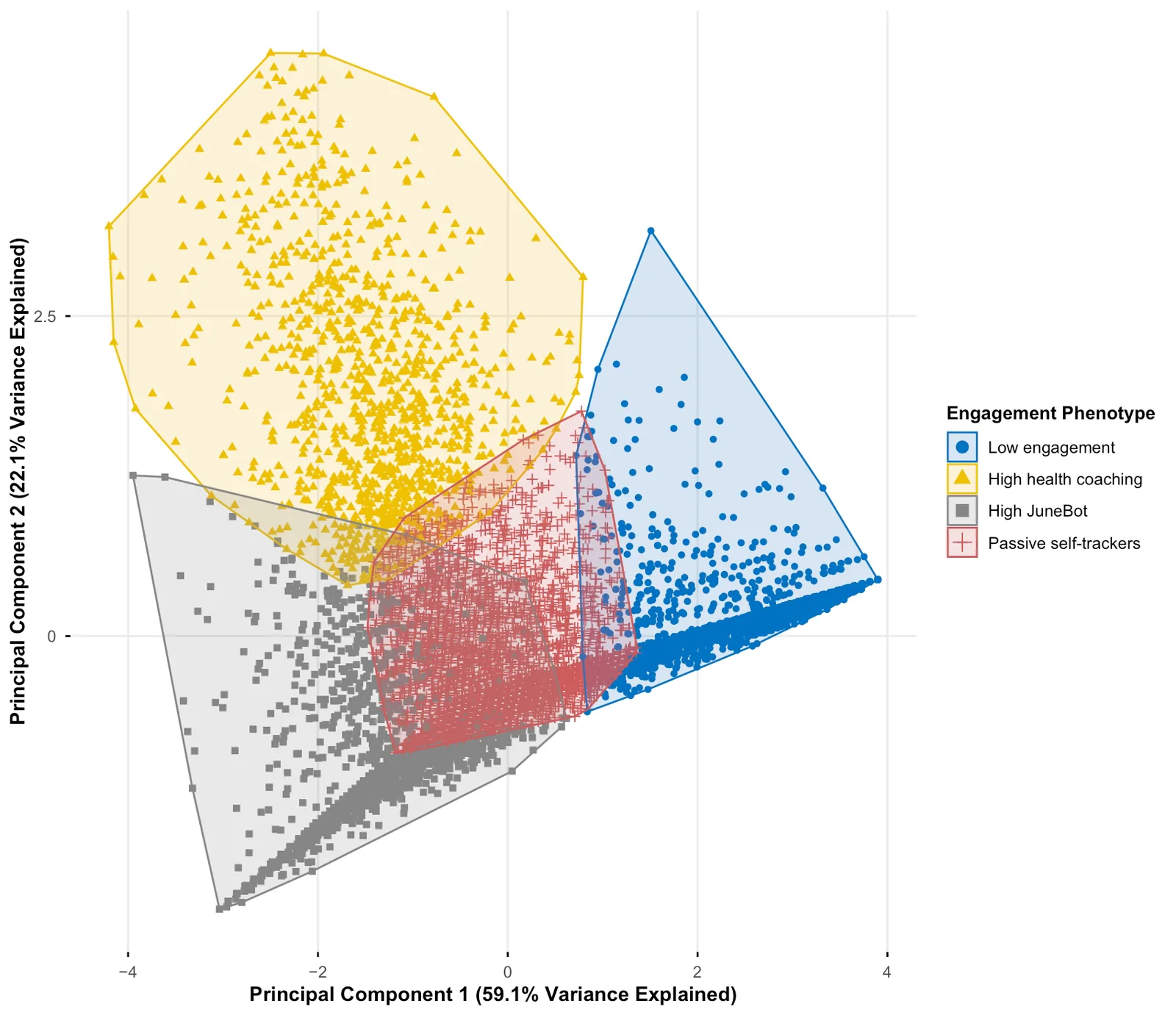
Two-dimensional principal component analysis projection of patient engagement phenotypes. Shaded convex hulls indicate the approximate regions occupied by each of the four K-means clusters in the space of the first two principal components. The horizontal and vertical axes represent the first and second principal components extracted from the four engagement metrics.

A post hoc principal component projection was used to visualise the clustering solution, with the first two components explaining 81.2% of the total variance across the four engagement metrics. Figure 2 shows the distribution of patients from the four clusters in this two-dimensional space, with substantial overlap between clusters in central regions.

Cluster-robustness analyses supported the 4-cluster engagement solution (Table 3). The gap statistic showed a clear improvement in fit up to K ≈ 4–5 with a plateau thereafter, and bootstrap resampling (B = 100) yielded high mean Jaccard similarities for all four clusters (0.99, 0.99, 0.99, 0.98), indicating excellent stability under resampling (Supplementary Figure S1, Tables S1 and S2).

### 3.2. Clinical Weight Loss Outcomes

Descriptive evaluation of the primary endpoint revealed distinct clinical trajectories across the engagement groups (Table 3). The unadjusted mean weight loss at 6 months was lowest in the non-engaged baseline cohort (11.7%; ±7.18%). Passive self-trackers achieved an unadjusted mean weight loss of 15.5% (±6.63%). The highest absolute unadjusted weight reductions were observed in both conversational cohorts, with the high health coaching group (16.4%; ±6.38%) and the High JuneBot group (16.4%; ±6.35%) recording identical mean clinical outcomes. In the low-engagement cluster, 81.0%, 59.1%, and 33.4% of patients achieved ≥5%, ≥10%, and ≥15% weight loss, respectively. Corresponding proportions were higher in all active engagement clusters: 95.2%, 85.8% and 60.2% in the high health coach cluster; 95.0%, 85.9% and 61.1% in the high JuneBot cluster; and 93.4%, 81.2% and 55.4% in the passive self-tracker cluster.

### 3.3. Unadjusted Pairwise Efficacy Comparisons (ANOVA/Tukey HSD)

A one-way ANOVA confirmed highly significant variances in unadjusted 6-month weight loss across the engagement groups (*p* < 0.001). Post hoc pairwise comparisons using Tukey’s Honest Significant Difference (HSD) test demonstrated significantly higher weight loss for all active engagement phenotypes over the baseline population.

Compared to the non-engaged group, mean weight loss was significantly higher in the passive self-trackers (+3.75%, 95% CI [3.27, 4.22], *p* < 0.001), the high health coaching group (+4.63%, 95% CI [3.98, 5.28], *p* < 0.001), and the high JuneBot group (+4.63%, 95% CI [4.03, 5.23], *p* < 0.001). Pairwise testing revealed no statistically significant difference in mean weight loss when comparing the high JuneBot group directly against the high health coaching group (+0.004%, 95% CI [−0.67, 0.68], *p* = 0.999). Both conversational phenotypes achieved significantly higher weight loss than passive tracking alone (*p* < 0.001).

### 3.4. Multivariate Regression Analysis

In the baseline-adjusted model including engagement clusters and pretreatment covariates (F = 50.53, adjusted R^2^ = 0.077, *p* < 0.001), the engagement prototypes remained strongly and significantly associated with 6-month percentage weight loss (Supplementary Table S4). Using the low-engagement group (Cluster 1) as the reference, adjusted differences in mean percentage weight loss were:

• Cluster 2 (high health coach engagement): β = 4.60%, SE = 0.26, t = 17.90, *p* < 0.001, 95% CI 4.10–5.11.
• Cluster 3 (high JuneBot engagement): β = 4.69%, SE = 0.24, t = 19.67, *p* < 0.001, 95% CI 4.22–5.16
• Cluster 4 (passive self-trackers): β = 3.71%, SE = 0.19, t = 20.03, *p* < 0.001, 95% CI 3.34–4.07

Younger age categories were associated with slightly greater weight loss compared to the 45–59 reference group (e.g., <30 years: β = 0.87%, SE = 0.23, *p* < 0.001), while male sex was associated with less weight loss than female sex (β = −0.94%, SE = 0.18, *p* < 0.001). Compared with a baseline BMI of 30–34.99 kg/m^2^, patients who were overweight (BMI < 30), had a BMI of 35–39.99, or a BMI ≥ 40 kg/m^2^ achieved progressively less weight loss (e.g., BMI ≥ 40: β = −2.22%, SE = 0.19, *p* < 0.001). Comorbidity count was not significantly associated with 6-month outcomes, whereas White ethnicity was associated with slightly greater weight loss (β = 0.92%, SE = 0.20, *p* < 0.001) and a history of previous GLP-1 RA use with less weight loss (β = −3.72%, SE = 1.12, *p* < 0.001). All VIFs were below 3 (maximum VIF = 2.4), suggesting that multicollinearity was not a major concern.

In an exploratory model that additionally included post-baseline variables (maximum tirzepatide dose, first-month weight-tracking category, any reported side effects, weight-loss plateau, and ≥5% weight loss at 3 months), these mid-program markers were also strongly associated with 6-month outcomes (Supplementary Table S5). In particular, achieving ≥5% weight loss within the first 3 months was associated with substantially greater 6-month percentage loss (β = 7.95%, SE = 0.17, *p* < 0.001), whereas experiencing a documented weight-loss plateau was associated with lower total 6-month weight loss (β = −2.91%, SE = 0.12, *p* < 0.001). Higher maximum tirzepatide doses (e.g., 12.5–15 mg) and being in higher first-month tracking categories were negatively associated with 6-month percentage loss compared with those of the reference groups.

## 4. Discussion

This retrospective per-protocol analysis of 9470 tirzepatide-treated patients in an app-mediated digital weight-loss service identified four naturally occurring digital engagement phenotypes and demonstrated that all active engagement profiles—passive self-tracking, high human health coach engagement, and high JuneBot engagement—were associated with greater 6-month percentage weight loss than a non-engaged phenotype. After adjustment for baseline demographic and clinical covariates, the conversational phenotypes (high health coach and high JuneBot engagement) remained strongly and significantly associated with greater 6-month percentage weight loss compared to the low-engagement reference group, while passive self-tracking showed a slightly smaller but still significant association. These mean differences were accompanied by higher proportions of patients reaching clinically recognised responder thresholds. In the low-engagement cluster, 81.0%, 59.1%, and 33.4% of patients achieved ≥5%, ≥10%, and ≥15% weight loss respectively. Corresponding proportions were higher in all active engagement clusters: 95.2%, 85.8%, and 60.2% in the high health coach cluster; 95.0%, 85.9%, and 61.1% in the high JuneBot cluster; and 93.4%, 81.2%, and 55.4% in the passive self-tracker cluster. Unadjusted comparisons yielded the same directional pattern, with clinically meaningful absolute differences between the low-engagement cohort and each active engagement group.

Prior studies exploring engagement and outcomes in digital weight-loss interventions have relied predominantly on binary engagement constructs or single-modality measures, limiting insight into how mixed or modality-specific behaviors relate to effectiveness. Johnson et al. [10] and similar work [11] advanced the literature by highlighting an engagement–effectiveness relationship but did not capture the multidimensional engagement patterns our clustering approach revealed. Our results extend this prior work by showing that distinct engagement archetypes, not just engaged versus non-engaged, map to different magnitudes of benefit and that automated conversational engagement can parallel human coaching when implemented within a tightly governed clinical ecosystem.

This study’s findings suggest that, within a real-world medicated DWLS, different modes of digital engagement are associated with different weight-loss outcomes. The similarity in baseline-adjusted effect sizes between high JuneBot engagement and high human health coach engagement is notable: a rule-based, non-agentic automated conversational tool used at scale was associated with mean weight loss that did not differ statistically from that observed in the high health coach group. This result aligns with the hypothesis that scalable digital conversational support can augment pharmacologic therapy [17] and may replicate some of the persistence support and behavioural reinforcement functions traditionally ascribed to human coaching.

However, the observational and retrospective nature of the analysis prohibits causal inference. It is possible that unmeasured patient characteristics, such as intrinsic motivation, digital literacy, socioeconomic factors not captured by remoteness or subscription tier, or prior experience with weight-loss programs, drive both engagement phenotype selection and therapeutic response. Similarly, the per-protocol sampling frame intentionally restricted the analytic cohort to patients who maintained medication orders and provided a 6-month weight, which may comprise individuals who are more motivated, have fewer adverse effects, or face fewer access barriers. Thus, the associations observed may overestimate effects that would be seen in an intention-to-treat population or in less adherent real-world cohorts.

It is also important to emphasize that a per protocol analysis fails to capture outcomes from the full cohort (i.e., patients who discontinued the program early, delayed medication orders or failed to submit data in the specified timeframe). However, previous studies have already established that 6-month attrition rates in digital chronic care services vary between roughly 40% and 49% [18], and between roughly 16% and 40% in real-world tirzepatide services [7,8]. The purpose of this study was to examine the relationship between program engagement and effectiveness for the subgroup of patients who adhere to a real-world medicated DWLS over 6 months.

### 4.1. Bridging Theoretical Behavioural Models and Real-World Engagement in Obesity Care

Achieving clinically meaningful weight loss (≥10% of initial body weight) is extremely challenging in overweight and obesity care. Previous studies of unmedicated interventions have revealed that roughly only 20% of individuals successfully achieve and maintain this level of weight loss for one year or longer [19]. While modern obesity medications such as tirzepatide have emerged as a promising solution to the global obesity challenge and are being increasingly prescribed, WHO and NICE stress that they are taken as a supplement to lifestyle interventions, including nutritional counseling, physical activity, and self-management support [6]. From a behavioral medicine perspective, some theoretical models emphasise an autonomously motivated patient who is able to independently sustain health-promoting behaviours over time [20,21]. However, evidence suggests that such autonomous self-regulation is difficult to achieve, since engagement, self-monitoring, and persistence commonly decline during ongoing interventions [22,23]. Our findings provide an interesting perspective on the gap between this theoretical ideal and the challenges in real-world practice. The autonomously engaged subgroup (passive self-trackers) in this study aligns with behavioural models that emphasise self-monitoring and autonomous engagement, and their outcomes (mean 15.5%) illustrate what can be achieved when engagement is sustained independently. However, we did not directly measure motivation or self-regulatory capacity, and these patients should not be interpreted as a normative ‘ideal’ against which others are judged. At the same time, the comparable outcomes observed among patients supported by health coaching and AI chatbots (both 16.4%) indicate that other subsets of patients benefit from additional support to maintain engagement. Health coaching and AI-based interventions should therefore not be viewed as alternatives to intrinsic motivation, but rather as mechanisms that support patients struggling with self-engagement while achieving similar weight-loss outcomes.

Collectively, these findings suggest that sustained engagement itself may be more important than the specific source from which it originates, as autonomous engagement, health coaching, and AI-supported engagement were all associated with clinically meaningful six-month weight-loss outcomes. This means that both internal and external sources of engagement can be effective and that interventions capable of sustaining engagement may help bridge the gap between patients who are self-motivated and those who may struggle to achieve durable weight-loss outcomes independently.

### 4.2. The Nuanced Role of Biometric Self-Monitoring

The clustering analysis indicated that sustained weekly weight tracking was a shared feature of the three higher-engagement phenotypes, consistent with prior work linking self-monitoring to better outcomes in behavioural interventions [3]. However, in the exploratory regression model that included first-month weight-tracking categories alongside other post-baseline variables, higher early tracking frequency was generally associated with lower 6-month percentage weight loss compared with logging only a baseline weight (e.g., 6–10, 11–15, and 16–25 entries all had negative coefficients). These associations must be interpreted cautiously in a per-protocol context: all patients in this cohort persisted with treatment and provided a 6-month weight, so first-month tracking frequency reflects patterns of data submission rather than overall program adherence. Without direct measures of anxiety, expectations, or distress, any interpretation of high early tracking as a proxy for psychological state remains speculative; alternative explanations, such as reverse causation (e.g., patients with a slower initial response tracking more frequently) or greater opportunity to capture short-term fluctuations, are also plausible. These findings underscore that tracking frequency should not be interpreted as a simple linear proxy for “better” engagement and that the prognostic role of early self-monitoring warrants further study.

### 4.3. Impact of Demographic and Clinical Milestones

In exploratory models that incorporated post-baseline markers, early clinical response exerted a large influence on 6-month outcomes. Achieving clinically significant weight loss (≥5%) within the first 3 months of the program was the single largest positive predictor in the model (β ≈ 7.95%, SE = 0.17, *p* < 0.001), suggesting that early biological response to tirzepatide is a critical driver of longitudinal success [24] and may act as a powerful motivational catalyst that reinforces programmatic adherence. Experiencing a documented weight-loss plateau during the program was, conversely, associated with substantially lower total 6-month weight loss (β ≈ −2.91%, SE = 0.12, *p* < 0.001). Conversely, maximum tirzepatide dose and first month track count were negatively associated with weight loss. Although the model controlled for clinical baseline factors such as comorbidity count and BMI, negative associations between weight loss and higher maximum tirzepatide doses likely reflect confounding by indication and delayed escalation among more difficult-to-treat patients. It needs to be stressed that all mid-program markers lie on the causal pathway, and are thus exploratory rather than reliable estimates of the total association between engagement phenotype and 6-month weight loss.

Demographically, younger age categories (under 30 and 30–44) and female sex were associated with small but statistically significant weight-loss advantages, aligning with broader real-world observations in medicated DWLS cohorts [8,25], while higher baseline BMI was associated with somewhat smaller percentage losses. Baseline comorbidity burden showed limited independent association with outcomes, and a history of previous GLP-1 RA use was associated with a somewhat lower 6-month percentage weight loss.

### 4.4. Public Health Implications

If reproduced in other settings and study designs, the equivalence between structured automated conversational support and human health coaching on short-term weight outcomes could have pragmatic implications for scaling comprehensive obesity care alongside GLP-1 RA access. Automated, rule-based systems may reduce the marginal cost of ongoing behavioral reinforcement [26], increase availability in regions with limited clinician capacity, and provide consistent persistence nudges without replacing essential clinical oversight. However, these potential benefits were not evaluated in this study and would require formal implementation and cost-effectiveness analyses. Importantly, the AI agent in this study was implemented with explicit non-medical, non-agentic guardrails and mandatory human escalation for flagged mental health or safety concerns. These safety constraints are essential if automated systems are to be broadly deployed as adjuncts to pharmacotherapy.

### 4.5. Strengths and Limitations

Strengths of this study include a large, clinically relevant sample of 9470 medication-adherent patients treated with tirzepatide within a real-world digital weight-loss service, the use of longitudinal, multidimensional engagement metrics and an unsupervised clustering approach that avoids arbitrary binary thresholds, and robust preprocessing and reproducibility steps (feature standardization, systematic K selection, multiple random starts with a fixed seed) together with a comprehensive regression adjustment set that reduces confounding from measured covariates. However, it also contained multiple limitations. Firstly, the retrospective, observational design precludes causal inference and leaves room for residual confounding by unmeasured factors such as motivation, socioeconomic status, digital literacy, and other behavioral traits that may determine both engagement and outcomes. Early response (e.g., ≥5% weight loss at 3 months) and plateau status were included only in exploratory models and should be interpreted as prognostic markers on the causal pathway rather than as baseline confounders in estimating the total association between engagement clusters and weight loss. Secondly, the per-protocol, complete-case sampling restricts the cohort to medication-adherent patients who provided 6-month weight date, introducing selection bias and limiting external validity to all initiators or less-adherent populations. Third, although we used standard quantitative criteria to guide clustering, several analytic decisions (choice of engagement variables, six-month aggregation window, and selection of K within a plausible range) involved investigator judgment, and the resulting phenotypes should be interpreted as one reasonable partition rather than the only possible representation of engagement behavior. Forth, the primary outcome depends on in-app or Bluetooth-linked weight entries that, despite verification processes, remain subject to measurement variability and potential reporting biases. Fifth, medication order numbers served as a proxy for medication persistence and data on actual medication administration were not available. Moreover, engagement variables and weight loss were measured over the same six-month period, so higher weight loss may have reinforced continued app use, messaging, and tracking, while poorer response or adverse effects may have reduced engagement. Accordingly, the clusters should be interpreted as concurrent six-month behavioral phenotypes associated with weight loss, rather than as definitively antecedent predictors of outcome. And finally, JuneBot operates under a highly restricted, rule-based, non-agentic communication framework. Therefore, these outcomes cannot be extrapolated to advanced, generative LLMs or autonomous AI clinical agents, which present entirely different safety, empathy, and operational profiles.

### 4.6. Implications for Future Research

Future work should pursue prospective and randomized designs to test whether routing patients to different support modalities (human coaching, structured automated conversational support, or hybrid models) causally improves outcomes and cost-effectiveness. Qualitative work exploring why patients select and sustain particular engagement patterns would strengthen external validity and implementation relevance. Studies incorporating richer socioeconomic measures, digital-literacy assessments, and validated measures of motivation and behavioral skills could help disentangle selection effects from intervention effects. Finally, longer-term follow-up is needed to assess maintenance of weight loss, safety, and the durability of engagement phenotypes.

## 5. Conclusions

In a large per-protocol cohort of tirzepatide-treated patients within a commercial app-mediated DWLS, naturally occurring digital engagement phenotypes were associated with differential 6-month weight-loss outcomes. After extensive covariate adjustment, both high human health coach engagement and high rule-based automated conversational engagement were independently associated with greater weight loss compared with a non-engaged phenotype, and these two conversational modalities demonstrated equivalent adjusted effects. These results are hypothesis-generating and suggest that scalable, safety-constrained automated conversational support could complement pharmacologic obesity treatment. Definitive causal conclusions require prospective, randomized evaluation and broader population sampling.

## Figures and Tables

**Table 1:** Baseline patient characteristics stratified by engagement cluster.

Variable	Cluster 1 Non-Engaged *N* = 1772	Cluster 2 High Health Coach Engagement *N* = 1141	Cluster 3 High Junebot Engagement *N* = 1472	Cluster 4 Passive Self- Trackers *N* = 5085	*p*-Value
Age category					<0.001
45–59	676 (38%)	457 (40%)	603 (41%)	1984 (39%)	
Under 30	210 (12%)	111 (9.6%)	153 (10%)	601 (12%)	
30–44	635 (36%)	373 (33%)	418 (28%)	1810 (35%)	
60+	251 (14%)	201 (17%)	298 (20%)	690 (13%)	
Sex at birth					<0.001
Female	1299 (73%)	1068 (94%)	1305 (89%)	4200 (83%)	
Male	473 (27%)	73 (6.4%)	167 (11%)	885 (17%)	
BMI category (kg/m^2^)					<0.001
<30	501 (28%)	189 (17%)	205 (14%)	947 (19%)	
30–34.99	761 (43%)	439 (38%)	570 (39%)	2036 (40%)	
35–39.99	290 (16%)	267 (23%)	346 (24%)	1115 (22%)	
40 and over	220 (12%)	246 (22%)	351 (24%)	987 (19%)	
Total comorbidities					<0.001
0	986 (56%)	364 (32%)	553 (38%)	2197 (43%)	
1	388 (22%)	271 (24%)	361 (25%)	1306 (26%)	
2	209 (12%)	221 (19%)	255 (17%)	820 (16%)	
3+	189 (11%)	285 (25%)	303 (21%)	762 (15%)	
Previous GLP-1 RA use	16 (0.9%)	3 (0.3%)	1 (<0.1%)	15 (0.3%)	<0.001

**Table 2:** Mean feature percentages by emergent engagement cluster.

Behavioral Metric	Cluster 1 Non-Engaged n = 1772	Cluster 2 High Health Coaching n = 1141	Cluster 3 High JuneBot n = 1472	Cluster 4 Passive Self-Trackers n = 5085
Weekly App Engagement (%)	30.0%	94.5%	95.0%	86.7%
Weekly Health Coach Messaged (%)	1.97%	50.9%	5.43%	5.25%
Weekly JuneBot Messaged (%)	5.46%	22.4%	46.2%	12.7%
Weekly Weight Track (%)	19.6%	86.2%	87.2%	78.8%

**Table 3:** Engagement cluster distribution.

Cluster Assignment	Patient Count (n)	Mean Weight Loss (%)	Standard Deviation (SD)	≥5% Weight Loss Achieved (%)	≥10% Weight Loss Achieved (%)	≥15% Weight Loss Achieved (%)
Cluster 1: Low engagement	1772	11.7%	7.18	81%	59.1%	33.4%
Cluster 2: High health coaching	1141	16.4%	6.38	95.2%	85.8%	60.2%
Cluster 3: High JuneBot	1472	16.4%	6.35	95%	85.9%	61.1%
Cluster 4: Passive self-trackers	5085	15.5%	6.63	93.4%	81.2%	55.4%

## Data Availability

De-identified aggregate data, a data dictionary, and the analysis code used in this study are available from the corresponding author upon reasonable request and subject to institutional and commercial data-protection policies. Individual-level raw data cannot be shared due to privacy and contractual restrictions. Synthetic or summarised datasets sufficient to reproduce the analytic pipeline may be provided on request, where permitted.

## Supplementary Materials

The following supporting information can be downloaded at https://www.mdpi.com/article/10.3390/medicina62091647/s1: Supplementary Figure S1. Gap statistic as a function of the number of clusters (K = 1–10). The statistic increases sharply up to K ≈ 4–5, with a plateau thereafter, and identifies K = 5 as optimal under the Tibshirani et al. criterion. Supplementary Table S1. Gap statistic values and standard errors for K = 1–10 from the clusGap procedure (B = 100 reference datasets). Supplementary Table S2. Mean Jaccard similarities from bootstrap cluster stability analysis (B = 100) of the 4 cluster k means solution. Higher values indicate greater stability under resampling. Supplementary Table S3. Median and IQR distributions of the four engagement metrics within each cluster. Supplementary Table S4. Multivariate regression analysis—baseline variables only. Supplementary Table S5. Multivariate regression analysis—baseline and post-baseline variables.

## Abbreviations

The following abbreviations are used in this manuscript:

ANOVA	Analysis of Variance
BMI	Body Mass Index
CI	Confidence Interval
DWLS	Digital Weight Loss Service
GIP	Glucose-Dependent Insulinotropic Polypeptide
GLP-1 RA	Glucagon-Like Peptide-1 Receptor Agonist
HSD	Honest Significant Difference (referring to Tukey’s post hoc test)
ITT	Intention-To-Treat
LLM	Large Language Model
MDT	Multidisciplinary Team
NICE	National Institute for Health and Care Excellence
NS	Not Statistically Significant
OLS	Ordinary Least Squares
PCA	Principal Component Analysis
PP	Per-Protocol
PSM	Propensity Score Matching
SD	Standard Deviation
SE	Standard Error
STROBE	Strengthening the Reporting of Observational Studies in Epidemiology
WHO	World Health Organization
WSS	Within-Cluster Sum of Squares
