# Supplementary material for: Digital Engagement Phenotypes and 6-Month Weight Loss in a Tirzepatide-Supported Digital Weight-Loss Program: A Retrospective Cohort Study
Source: Medicina (Kaunas). 2026 Aug 27;62(9):1647. doi: 10.3390/medicina62091647 (PMC13609581; doi:10.3390/medicina62091647)
Supplement: Supplementary file 1 [file medicina-62-01647-s001.zip › medicina-4430847-supplementary.pdf]

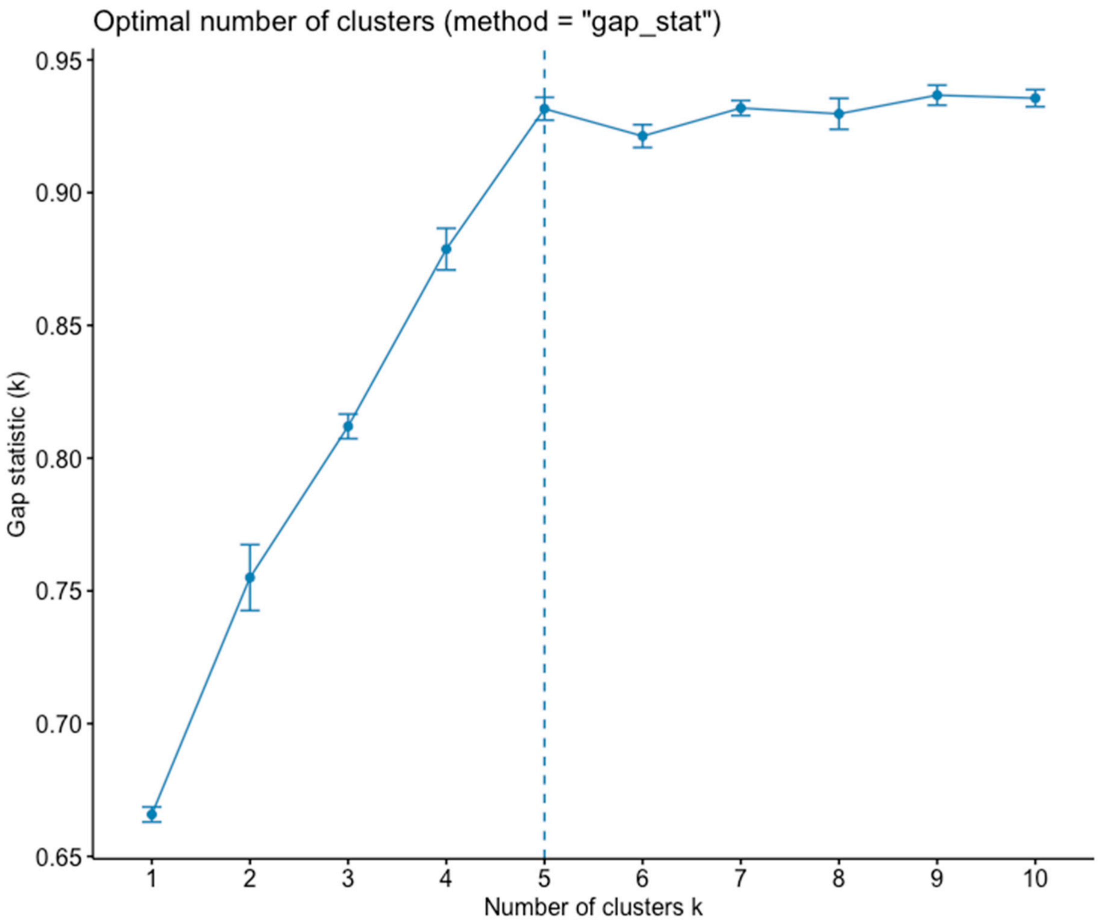

Supplementary Figure S1. Gap statistic as a function of the number of clusters (K = 1–10). The statistic increases sharply up to K ≈ 4–5, with a plateau thereafter, and identifies K = 5 as optimal under the Tibshirani et al. criterion.

Supplementary Table S1. Gap statistic values and standard errors for K = 1–10 from the clusGap procedure (B = 100 reference datasets).

K	logW	E.logW	Gap	SE.sim
1	8.66994	9.33573	0.66579	0.00283
2	8.39110	9.14610	0.75501	0.01242
3	8.23158	9.04355	0.81196	0.00463
4	8.07371	8.95244	0.87872	0.00784
5	7.94512	8.87671	0.93159	0.00430
6	7.87956	8.80085	0.92129	0.00430
7	7.81419	8.74605	0.93186	0.00284

8	7.76211	8.69179	0.92968	0.00584
9	7.72100	8.65770	0.93670	0.00377
10	7.69048	8.62607	0.93558	0.00322

Supplementary Table S2. Mean Jaccard similarities from bootstrap cluster-stability analysis (B = 100) of the 4-cluster k-means solution. Higher values indicate greater stability under resampling.

Cluster (label)	Mean Jaccard similarity
1 – Low engagement	0.9901
2 – High health coaching	0.9920
3 – High JuneBot	0.9919
4 – Passive self-trackers	0.9844

Supplementary Table S3. Median and IQR distributions of the four engagement metrics within each cluster.

Cluster Assignment	n	App engagement, median % (IQR)	Health coach engagement, median % (IQR)	Junebot engagement, median % (IQR)	Weight tracking, median % (IQR)
**Cluster 1:** Low-engagement	1,772	29.6 (11.1-48.1)	0 (0-2)	3.7 (0-7.4)	14.8 (3.7-33.3)
**Cluster 2:** High health coaching	1,141	96.4 (92.6-100)	48.4 (37-63)	18.5 (11.1-29.6)	89.3 (78.6-96.3)
**Cluster 3:** High JuneBot	1,472	96.4 (92.6-100)_	3.7(0-7.41)	46.2 (33.3-52.3)	92.6 (81.5-96.3)
**Cluster 4:** Passive self-trackers	5,085	88.9 (77.8-96.3)	3.7 (0-10.7)	11.1 (7.4-18.5)	81.5 (66.7-92.6)

Supplementary Table S4. Multivariate regression analysis – baseline variables only.

Predictor Variable	Estimate (β)	SE	T-statistic	P-value	95% CI
(Intercept)	11.70	0.28	42.60	< .001	[11.10, 12.20]
Engagement Prototype (Ref: Cluster 1: Non-Engaged)					
Cluster 2	4.60	0.26	17.90	< .001	[4.10, 5.11]
Cluster 3	4.69	0.24	19.70	< .001	[4.22, 5.16]
Cluster 4	3.71	0.19	20.00	< .001	[3.34, 4.07]

Age Category (Ref: 45–59)					
Age: Under 30	0.87	0.23	3.73	< .001	[0.41, 1.33]
Age: 30–44	0.65	0.16	4.01	< .001	[0.33, 0.96]
Age: 60+	-0.18	0.21	-0.87	.383	[-0.59, 0.23]
Sex at Birth: Male	-0.94	0.18	-5.12	< .001	[-1.31, -0.58]
BMI Category (kg/m²) (Ref: 30–34.99)					
BMI: <30	-1.10	0.19	-5.74	< .001	[-1.47, -0.72]
BMI: 35–39.99	-0.79	0.18	-4.33	< .001	[-1.15, -0.43]
BMI: 40 and over	-2.22	0.19	-11.50	< .001	[-2.59, -1.84]
Total Comorbidities (Ref: 0)					
Comorbidity Count: 1	0.19	0.17	1.11	.265	[-0.15, 0.53]
Comorbidity Count: 2	-0.25	0.20	-1.21	.228	[-0.64, 0.15]
Comorbidity Count: 3+	-0.25	0.21	-1.20	.232	[-0.65, 0.16]
Ethnicity: White (vs non-white)	0.92	0.20	4.53	< .001	[0.52, 1.31]
Previous GLP-1 Use (Yes)	-3.72	1.12	-3.33	< .001	[-5.92, -1.53]

Supplementary Table S5. Multivariate regression analysis – baseline and post-baseline variables.

Predictor Variable	Estimate (β)	Standard Error	t-statistic	P-value	95% CI
**(Intercept)**	9.59	0.36	26.84	< .001	[8.89, 10.29]
**Engagement Prototype (Ref: Cluster 1: Non-Engaged)**					
Cluster 2	2.59	0.27	9.65	< .001	[2.06, 3.11]
Cluster 3	2.76	0.25	11.00	< .001	[2.27, 3.25]
Cluster 4	2.04	0.21	9.71	< .001	[1.63, 2.46]
**Age Category (Ref: 45–59)**					
Age: Under 30	0.66	0.20	3.24	.001	[0.26, 1.05]
Age: 30–44	0.31	0.14	2.23	.026	[0.04, 0.58]
Age: 60+	-0.03	0.18	-0.19	.847	[-0.39, 0.32]
Age: Unknown	0.70	0.80	0.88	.379	[-0.86, 2.27]
Sex at Birth: Male	-0.70	0.16	-4.26	< .001	[-1.02, -0.38]
**BMI Category (kg/m²) (Ref: 30–34.99)**					

BMI: Overweight	-0.58	0.17	-3.46	< .001	[-0.90, -0.25]
BMI: 35–39.99	-0.63	0.16	-3.95	< .001	[-0.94, -0.32]
BMI: 40 and over	-1.75	0.17	-10.38	< .001	[-2.08, -1.42]
**Total Comorbidities (Ref: 0)**					
Comorbidity Count: 1	-0.02	0.15	-0.11	.914	[-0.31, 0.28]
Comorbidity Count: 2	-0.18	0.18	-1.03	.303	[-0.53, 0.16]
Comorbidity Count: 3+	-0.35	0.18	-1.93	.054	[-0.70, 0.01]
**Ethnicity (Ref: Non-White)**					
Ethnicity: White	0.38	0.18	2.14	.032	[0.03, 0.72]
**Prior Treatment History**					
Previous GLP-1 RA Use	-1.02	0.97	-1.05	.295	[-2.92, 0.89]
**Max tirzepatide dose (Ref: 7.5mg)**					
Max Dose: 10 mg	-0.51	1.28	-0.40	.689	[-3.02, 2.00]
Max Dose: 12.5 mg	-3.53	1.15	-3.07	.002	[-5.78, -1.28]
Max Dose: 15 mg	-3.44	0.56	-6.16	< .001	[-4.53, -2.34]
Max Dose: 2.4 mg	-2.73	2.02	-1.35	.176	[-6.69, 1.23]
Max Dose: 2.5 mg	-1.34	0.30	-4.42	< .001	[-1.94, -0.75]
Max Dose: 5 mg	-0.61	0.15	-3.94	< .001	[-0.91, -0.31]
**First-Month Weight-Tracking Count (Ref: Baseline only)**					
Tracking Count: 2–5	-0.81	0.33	-2.45	.014	[-1.46, -0.16]
Tracking Count: 6–10	-1.32	0.35	-3.79	< .001	[-2.00, -0.64]
Tracking Count: 11–15	-1.72	0.37	-4.62	< .001	[-2.45, -0.99]
Tracking Count: 16–25	-1.17	0.38	-3.11	.002	[-1.91, -0.43]
Tracking Count: > 25	-0.40	0.38	-1.05	.295	[-1.14, 0.35]

**Mid-Program Characteristics & Outcomes**					
Any Side Effects	0.06	0.13	0.47	.639	[-0.20, 0.33]
Weight Loss Plateau	-2.91	0.12	-23.51	< .001	[-3.15, -2.67]
Significant 3-Month Weight Loss (≥ 5%)	7.95	0.17	45.91	< .001	[7.62, 8.29]
